# Location, location, location: survival of Antarctic biota requires the best real estate

**DOI:** 10.1098/rsbl.2022.0590

**Published:** 2023-03-22

**Authors:** Mark I. Stevens, Andrew N. Mackintosh

**Affiliations:** ^1^ Securing Antarctica's Environmental Future, Earth and Biological Sciences, South Australian Museum, SA 5000, Australia; ^2^ School of Biological Sciences, University of Adelaide, SA 5005, Australia; ^3^ Securing Antarctica's Environmental Future, School of Earth, Atmosphere and Environment, Monash University, Melbourne, VIC 3800, Australia

**Keywords:** springtails, ice-free, glacial refuge, cosmogenic dating, *nunatak*, ice sheet

## Abstract

The origin of terrestrial biota in Antarctica has been debated since the discovery of springtails on the first historic voyages to the southern continent more than 120 years ago. A plausible explanation for the long-term persistence of life requiring ice-free land on continental Antarctica has, however, remained elusive. The default *glacial eradication* scenario has dominated because hypotheses to date have failed to provide a mechanism for their widespread survival on the continent, particularly through the Last Glacial Maximum when geological evidence demonstrates that the ice sheet was more extensive than present. Here, we provide support for the alternative *nunatak refuge* hypothesis—that ice-free terrain with sufficient relief above the ice sheet provided refuges and was a source for terrestrial biota found today. This hypothesis is supported here by an increased understanding from the combination of biological and geological evidence, and we outline a mechanism for these refuges during successive glacial maxima that also provides a source for coastal species. Our cross-disciplinary approach provides future directions to further test this hypothesis that will lead to new insights into the evolution of Antarctic landscapes and how they have shaped the biota through a changing climate.

## 1. Ancient relicts or recent colonizers?

The presence of biota, such as springtails (Arthropoda: Collembola) that inhabit Antarctic ice-free areas year-round, has troubled biologists for more than 120 years since their discovery during the first historic voyages. The first springtails collected were during the Belgian Antarctic Expedition (1897–1899) from Harry Island (Antarctic Peninsula) [1,2], while Herluf Klövstad collected the first springtails from continental Antarctica on the northern coast of Victoria Land (Ross Sea Region) during the Southern Cross British Antarctic Expedition (1898–1900) [3]. Springtails are now known to exist widely in ice-free areas across Antarctica [4–7]. As more species were discovered, researchers began to question whether they colonized since the Last Glacial Maximum (LGM; 26–19 kya [8]) when it was assumed that most of Antarctica was ice covered. The alternative, and less favoured, scenario was that they were relict species that survived in isolation for millions of years from pre-glacial times [9–13] when they shared the continent with a suite of, now extinct, biota that included *Nothofagus*-herb tundra, weevils, flies, freshwater fish, gastropods, bivalves and ostracods [14–19]. Two issues have hampered our ability to adequately evaluate these competing theories of introduction versus long-term survival; first, that the morphological characters used to define species have erroneously provided an illusion that some are cosmopolitan (e.g. [4,5]), and second, that early ice sheet reconstructions suggested that Antarctica was covered by a significant ice sheet at the LGM (e.g. [20]), which presumably subsumed most currently ice-free terrain. These issues led to the assumption that biota colonized the continent during interglacial periods when ice retreat provided sufficient ice-free habitats; the assumption of *glacial eradication* came to dominate (e.g. [21]).

More recently, the alternative *nunatak refuge* hypothesis—that the survival of the vast majority of biota during glacial maxima occurred on terrain that protruded through the ice sheet (nunataks) with sufficient relief to have remained ice-free—has gained support based only on the unique assemblage of biota present [9,10,22]. Despite this support, it has remained perplexing because it fails to account for the vast majority of biota that today resides in low-elevation coastal margins of Antarctica [22]. In an attempt to reconcile the presence of Antarctic species along or near the Antarctic coastal margins, *geothermal activity* was proposed as a solution [23]. Here, we detail limitations of this hypothesis to account for the most biodiverse regions in Antarctica and provide support for the alternative *nunatak refuge* hypothesis. Importantly, we have compiled support by using an increased understanding from the *combination of biological and geological evidence*, and we detail a mechanism that provides an essential source for coastal species.

## 2. A framework for the survival of Antarctic biota during glacial maxima

### (a) Geothermal glacial refugia hypothesis

**Figure 1.**
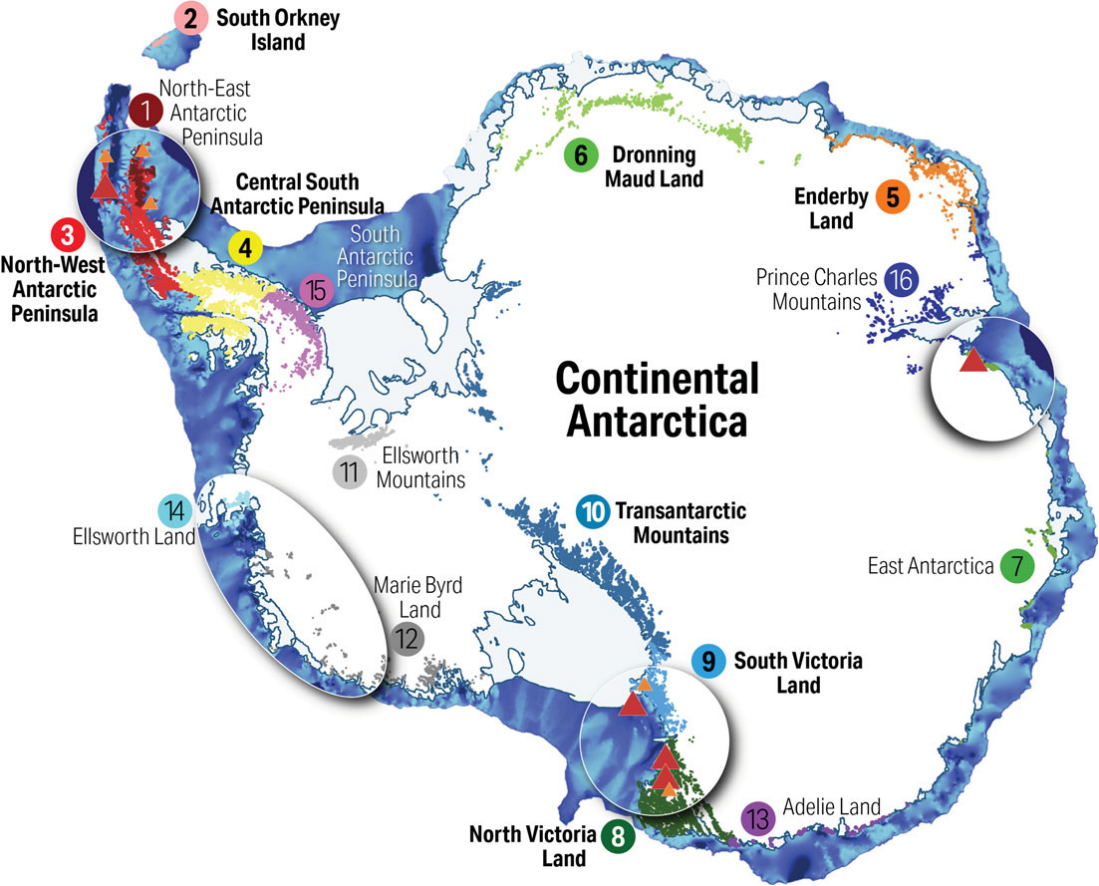
All terrain ice-free today shown as colour-shaded regions that represent the 16 currently recognized Antarctic Conservation Biodiversity Regions (ACBRs), where those labelled in bold text contain springtail species that accounts for 82% of the total ACBR area [25]. Springtail species occur in two of the four volcanic regions (within 100 km) highlighted by ellipses, where small (orange) and large (red) geothermal-specific sites are indicated (adapted from [24]). Blue shade around Antarctica matches the current below sea level shown in figure 2*a*. Map created using Quantarctica [26] in QGIS ver. 3.22.7 [27] with our compiled data files [28].

The most extensive reviews exploring the survival of Antarctic biota during glacial maxima, although acknowledging the unique endemic species that exist on the continent, struggle to explain the majority of biota that is currently found near the *coastal regions of Antarctica* [11–13,22]. In an attempt to reconcile the persistence of Antarctic coastal biota, one hypothesis suggested by Convey & Lewis Smith [23] and later tested by Fraser *et al*. [24] postulated that *geothermal activity* provides a mechanism to maintain ice-free land near coastal margins during glacial maxima. For *geothermal activity* to provide a plausible mechanism maintaining ice-free coastal refuges, it would require there to be sufficient sites throughout Antarctica where regionally isolated endemic species would have persisted across the LGM and into the present. Fraser *et al*. [24], using a 100 km radius, showed that the survival of springtails (and other biota) occurs today in the vicinity of some geothermal sites on the South Shetland Islands and northern Antarctic Peninsula (figure 1; electronic supplementary material, figure S1), which were also recently suggested as playing a role in the persistence of springtails across the LGM and prior [29]. However, these species have much wider distributions, including the Antarctic Peninsula and offshore islands [6]. Likewise, the proximity to geothermal sites of three (of eight) species in Victoria Land is at least within 100 km (figure 1; electronic supplementary material, figures S4 and S5). However, for springtails that are not found outside Antarctica [6] and predominantly are distributed along coastal margins, the vast majority *do not overlap with any geothermal site* (figure 1; electronic supplementary material, figures S1–S7). Geothermal activity and other potential refugia (see [22]), while perhaps locally important in a few specific cases, do not provide an explanation for broader species survival in coastal regions at the LGM or through previous glacial maxima.

### (b) Nunatak refuge hypothesis

#### (i) Geological evidence

**Figure 2.**
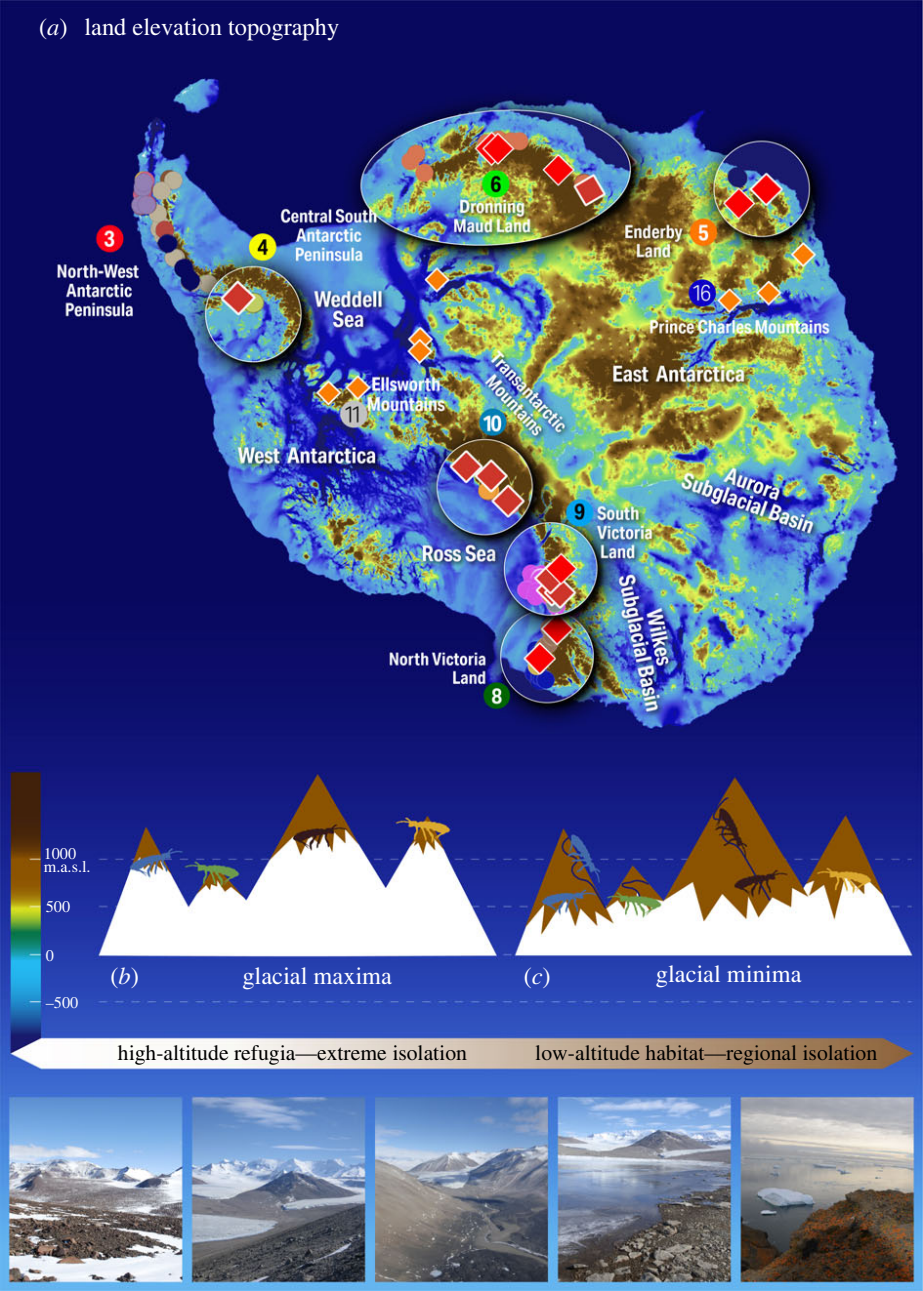
(*a*) Distribution of springtail species shown as coloured dots with ellipses indicating short-range endemic species, overlaid on the land elevational topography of Antarctica (blue = below sea level, green/yellow/brown = above sea level, see key in (*b*)). The presence of every known endemic springtail species on the continent and central Antarctic Peninsula is within 100 km of LGM ice-free refuges indicated by cosmogenic dating (red diamonds) and these occur in six ACBRs, while potential ice-free refugia without springtails occur in three ACBRs (orange diamonds) (compiled from https://www.ice-d.org/). (*b*) Survival of springtails (colours represent isolated species) in ice-free terrain shift with glacial margins (foreland) as ice expands during glacial maxima. (*c*) Dispersal of springtails during glacial minima (interglacial) shift with the glacial foreland as lower altitude ice-free land becomes available; biota disperse into low-elevation habitats into coastal regions. Map and distance measurements performed using Quantarctica and land elevational topography created using BedMachine [52], in QGIS with our compiled data files [28].

For the *nunatak refuge* hypothesis to be robust, we need to explore if variations in ice sheet thickness across Antarctica during glacial maxima allowed for widespread ice-free habitats to be maintained. Modern ice sheet reconstructions based on both onshore and offshore geological evidence, as well as dating of these features shows that the ice sheet at the LGM expanded onto the continental shelf and in coastal regions it thickened by hundreds of metres [30,31], with the greatest increases (greater than 1 km) observed in West Antarctica [32,33]. Cosmogenic isotope dating is uniquely suited to Antarctic environments [34] where geological dating of glacial deposits on the flanks of Antarctic nunataks indicates in many cases mountain slopes and some moraines remained uncovered during the LGM [35–45]. Furthermore, cosmogenic isotope data from regions with extensive datasets and/or consistent findings from multiple nuclides (see electronic supplementary material) indicate that persistent ice-free conditions have likely existed since at least the LGM and in many cases much longer [35,37,38,40,43,46–51] (figure 2*a*; electronic supplementary material, figures S1–S7). Most of these regions harbour short-range endemic biota, including springtails (figure 2*a*). In some locations, such as Victoria Land, Transantarctic Mountains and Dronning Maud Land, there is evidence of ice-free conditions persisting for millions of years [40,43,46]. In Victoria Land, low-altitude moraines provide an additional habitat [4], and such features may persist for thousands of years, particularly when they are associated with long-standing nunataks [53]. By contrast, despite evidence for ice-free nunataks [33,38,40,49–51], springtails appear to be entirely absent from marine-based sectors of the ice sheet (West Antarctica, the Weddell Sea sector, and Wilkes and Aurora subglacial basins) where the ice is grounded below sea level [54] (figure 2*a*; electronic supplementary material, figures S3, S6 and S7). Such marine-based glaciers are known to be highly dynamic, substantially expanding and contracting during glacial-interglacial cycles (for example, in East Antarctica, Marie Byrd Land and Ellsworth Land [33,55–57]) providing limited long-term refuge for biota.

### (ii) Biological evidence

For Antarctic biota, there is a growing body of molecular evidence revealing long-term isolation and persistence of short-range endemic species on the Antarctic continent [7,58–64]. For springtails that are a dominant driver for biodiversity patterns across the Antarctic realm [25] (figure 1), data show that these endemic species were likely present in ice-free refuges for at least 15–12 Ma [17,46,58]. Endemism is present in most sectors of the continent [6,7], and this is supported by geological dating indicating long-term ice-free conditions at sites (figure 2*a*; see also electronic supplementary material, file). *So how does this explain species presence today in coastal habitats?* Clues to this remarkable survival come from well-known alpine and polar studies (e.g. [65–68]). In alpine and polar regions, the greatest biodiversity is found living in or near glacial forelands [65–71] and studies have shown that *biota occupy this glacial foreland ecosystem as it ‘shifts’ adjacent to glacial margins* as they expand or contract [49,65,66,68,71,72]. This association adjacent to modern ice has also been identified as one of the most biodiverse ecological zones in Antarctica, despite the proximity of biota to a much-expanded ice front during glacial maxima [69–71]. This scenario is illustrated in figure 2*b*, where, during glacial maxima, species contract to small pockets of favourable habitat (such as a glacial foreland ecosystem or analogous moraine ecosystems) in terrain that protruded above or on the modern ice sheet (figure 2*b*). During an interglacial, species disperse down slopes and valleys with the glacial foreland that shifts with glacial margins as the ice retreats (figure 2*c*), with dispersal also potentially being assisted by meltwater (that is well known as a dispersal vector, e.g. [73]) spreading biota towards coastal areas as they become ice-free. Today, the presence of every known endemic springtail species on the continent and central Antarctic Peninsula is within 100 km of known LGM ice-free refuges indicated by cosmogenic dating (figure 2*a*; electronic supplementary material, figures S1–S7). The converse is also true; springtails are absent from extensive ice-free regions (see figures 1 and 2*a*), likely because these regions do not contain terrain with appropriate environments to serve as refuges when lowland ice expanded during glacial maxima.

## 3. New directions

Our suggested framework provides the necessary way forward to *test predictions using an evidence-based approach*. We posit that Antarctica's nunataks provide the most likely refuge for the long-term survival of species, and this explains the patterns of isolated short-range endemic species we find today, where there is an increasing body of evidence that none are shared between regions and some regions are entirely lacking in species. The *nunatak refuge hypothesis* also explains how species can be present during interglacials in coastal areas adjacent to their refugia—they do so by shifting up and down slopes within glacial foreland ecosystems.

Going forward, *cross-disciplinary collaborations* can provide a more complete picture of the evolution of Antarctic landscapes and the biota they harbour, but this requires cosmogenic isotope dating specifically targeted towards identifying ice-free terrain at the LGM or older (e.g. [40,43,45]), integrated with biological investigations that unequivocally define connectivity versus isolation (e.g. [7,59,61,62,64,74,75]) to identify broader refugial sites. While we have well-synchronized geochronology with springtail presence in ice-free conditions in Victoria Land, the Transantarctic Mountains and Dronning Maud Land (figure 2*a*), there are three specific tests that can be carried out to further evaluate the *nunatak refuge hypothesis*: (1) the Antarctic Peninsula has well-documented springtail records, but their provenance is uncertain [6,29] and age control on LGM and earlier landscapes is sparse [38]—further biological (using robust molecular dating, e.g. [76]) and geochronology are required to evaluate whether nunataks remained ice-free; (2) regions where geochronology indicates that nunataks likely remained ice-free at the LGM (for example in the Prince Charles Mountains; [40,77]), but where, to date, evidence for springtail presence remains equivocal albeit for recent environmental DNA signatures from soils [78]—such biological signals warrant further exploration and (3) greater focus on regions where the geochronology is suggestive of ice-free nunataks at the LGM [35,36,47–51] and biological investigations are limited or yet to be done, for example, in Enderby Land, Ellsworth Mountains and Transantarctic Mountains (figure 2*a*).

## 4. Conclusion

We have described a mechanism that allowed for springtails to survive in isolation in refugia across the LGM in ice-free habitats. Support for long-term continuity across Antarctic ice-free terrain is provided by geological dating of glacial deposits since the ice sheet first formed (approx. 34 Ma; [79–81]). More recently, molecular data have revealed that species found on the continent are a suite of unique locally endemic survivors from at least the last 15–12 Ma [7,56,58] when ice sheet thickening appears to have reached its maximum [17,46,82]. Together, these data highlight striking levels of endemism on the continent revealing biodiversity patterns in ice-free Antarctica that contributes significant insights to the now widely accepted ‘Antarctic Continental Biodiversity Regions' (ACBRs) [25] (figure 1).

Recent modelling of snow and ice melt indicates that current ice-free regions in Antarctica may expand up to 25% within the twenty-first century [83]. If these projections are correct, Antarctica will be changed forever with anthropogenic climate change, and endemic springtails once regionally isolated may no longer need refugia that have served them well for millions of years. How these once isolated communities cope with a vastly changing landscape with biotic interactions not experienced for millions of years remains largely unknown.

## Data Availability

Supplementary files, including all data files we used in QGIS for springtail records, geothermal and geochronological sites shown in figures 1 and 2 are available from the Dryad Digital Repository: https://doi.org/10.5061/dryad.zw3r228bx [28]. The data are provided in the electronic supplementary material [84].
